# *Slim-Filter*: an interactive windows-based application for illumina genome analyzer data assessment and manipulation

**DOI:** 10.1186/1471-2105-13-166

**Published:** 2012-07-16

**Authors:** Georgiy Golovko, Kamil Khanipov, Mark Rojas, Antonio Martinez-Alcántara, Jesse J Howard, Efren Ballesteros, Sharu Gupta, William Widger, Yuriy Fofanov

**Affiliations:** 1 Center for BioMedical and Environmental Genomics, University of Houston, Houston, TX, USA; 2 Department of Computer Science, University of Houston, Houston, TX, 77204, USA; 3 Department of Biology and Biochemistry, University of Houston, Houston, TX, 77204, USA

## Abstract

**Background:**

The emergence of Next Generation Sequencing technologies has made it possible for individual investigators to generate gigabases of sequencing data per week. Effective analysis and manipulation of these data is limited due to large file sizes, so even simple tasks such as data filtration and quality assessment have to be performed in several steps. This requires (potentially problematic) interaction between the investigator and a bioinformatics/computational service provider. Furthermore, such services are often performed using specialized computational facilities.

**Results:**

We present a Windows-based application, *Slim-Filter* designed to interactively examine the statistical properties of sequencing reads produced by *Illumina Genome Analyzer* and to perform a broad spectrum of data manipulation tasks including: filtration of low quality and low complexity reads; filtration of reads containing undesired subsequences (such as parts of adapters and PCR primers used during the sample and sequencing libraries preparation steps); excluding duplicated reads (while keeping each read’s copy number information in a specialized data format); and sorting reads by copy numbers allowing for easy access and manual editing of the resulting files. *Slim-Filter* is organized as a sequence of windows summarizing the statistical properties of the reads. Each data manipulation step has roll-back abilities, allowing for return to previous steps of the data analysis process. *Slim-Filter* is written in C++ and is compatible with *fasta*, *fastq*, and specialized *AS* file formats presented in this manuscript. Setup files and a user’s manual are available for download at the supplementary web site ( https://www.bioinfo.uh.edu/Slim_Filter/).

**Conclusion:**

The presented Windows-based application has been developed with the goal of providing individual investigators with integrated sequencing reads analysis, curation, and manipulation capabilities.

## Background

**Figure 1:**
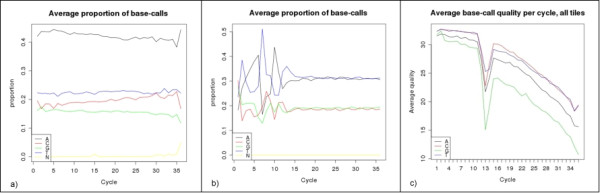
**Typical biases in the position-by-position proportion of nucleotides and their average quality across reads produced by an Illumina GAIIx instrument: a) nucleotide *A *present in higher proportion across all positions; b) first 10+ positions are biased to adapter sequences; c) the overall average quality of the nucleotide *G *is lower across all positions and a sharp quality drop is observed near position 13.**

Next Generation Sequencing instruments such as the *Illumina Genome Analyzer* (Illumina Inc.), *SOLiD* (Life Technologies), 454 (Life Sciences), and Ion PGM™ and Ion Proton™ (Life Technologies) are able to produce dozens of gigabases of sequencing data per week. The *Illumina Genome Analyzer* and *SOLiD* platforms produce large data files containing relatively short subsequences (*reads*) of equal size, usually 30–120 bases long. Each step in the sequencing process, such as sample preparation, library generation and base calling, can introduce significant biases and sequencing errors. For example: scanner calibration errors can cause one or more specific nucleotides to appear at deviated frequencies in the sequencing dataset (Figure 1a). A high concentration of adapters or PCR primers presented during sample preparation can cause biases at the beginning of the reads (Figure 1b). Additionally, physical disturbances (such as vibration of the instrument) during the sequencing process can lead to a drop in the quality of one or more sequencing cycles (Figure 1c).

A variety of platform-independent, as well as instrument-specific, applications have been developed to identify such biases and errors in the sequencing data [1-4]. Reads manipulation tools have also been developed to perform tasks such as sorting, trimming, filtering, eliminating duplications, and reads curation [5-9].

A major inconvenience however, is that each phase in the data manipulation process and in the recalculation of the statistical characteristics of the datasets requires separate applications and must be performed as a series of disconnected steps. The presented interactive Windows-based application (*Slim-Filter*) performs a variety of data manipulation tasks while simultaneously monitoring the statistical properties of the dataset and allowing users to save and/or undo (roll-back) each step of the analysis. Also available is a Linux version that has better performance, but is lacking the interactive and visual capabilities of the Windows-based version. *Slim-Filter* is compatible with standard *Illumina Genome Analyzer* file formats (such as *fasta* and *fastq*), as well as a presented new *AS (Array Subsequences)* format designed to store only unique reads and their copy numbers in descending order (by copy number).

## Implementation

*Slim-Filter* for Windows is implemented in VC++ and supports all Windows 64 bit operating systems. Plots and diagrams that represent statistical properties exploit the *.Net* library and require a Microsoft.NET Framework 3.5 (or a more recent version). The Linux version of *Slim-Filter* provides similar functionality, but is implemented as a one-step command line application. *S*etup files, example data, file format descriptions, and the user manual are available on the supplementary website ( http://www.bioinfo.uh.edu/Slim_Filter).

### Windows-based interface

**Figure 2:**
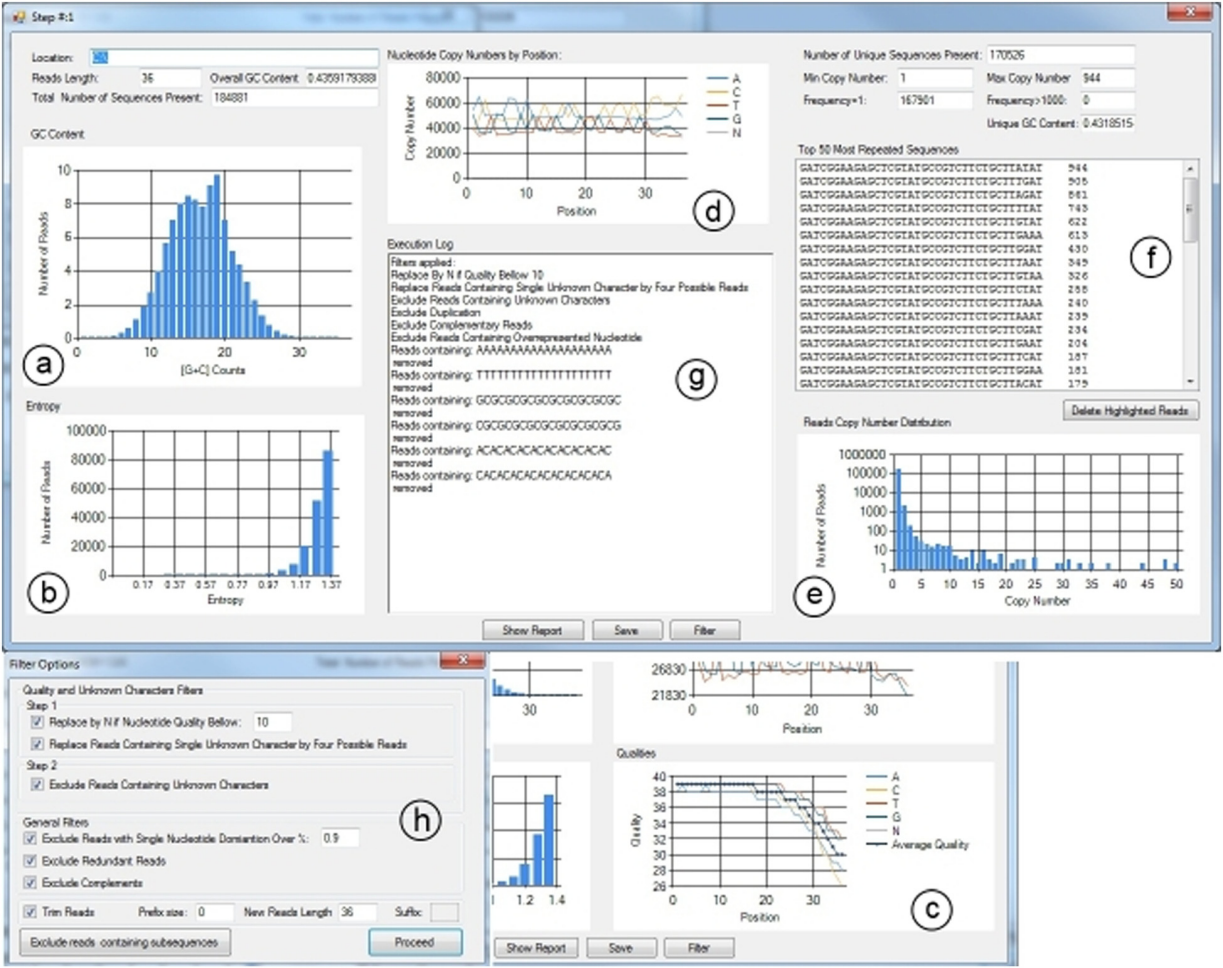
Statistical characteristics of the set of reads: a) GC content distribution, b) entropy of individual reads distribution, c) average position-by-position nucleotide quality, d) position-by-position proportion of each nucleotide, e) reads copy number distribution, f) list of 50 most frequent reads, g) summary of the previously applied steps, h) filter options menu.

*Slim-Filter* for Windows is organized as a chain of data manipulation steps which can contain several tasks. The resulting new set of reads can be saved in *fasta*, *fastq,* or *AS* formats. Corresponding windows summarize statistical properties of the resulting reads (Figure 2). The sequential windows containing statistical summaries for each data manipulation step can remain open, allowing for a before and after comparison of the datasets. *Slim-Filter* allows the user to return (roll-back) to any of the previous steps of the analysis and proceed with a different set of tasks and/or task parameters. The log of the step-by-step statistical analysis and the history of data manipulation is stored in a report text file.

### Data manipulation

*Slim-Filter* can perform eleven different data manipulation tasks. If the input data is available in the *fastq* file format, the user can (1) exclude reads with a quality score below a user-defined threshold, or (2) replace each low quality nucleotide with the “unknown nucleotide” symbol *N*. The other nine tasks do not require nucleotide quality information and can be performed on data in the *fastq, fasta* and *AS* formats. Each read that contains a single unknown nucleotide can be (3) replaced by four alternative reads where the *N* symbol is replaced by each of the four possible nucleotides (*A, C, T, G*). This tactic allows for the “recovery” of data from damaged sequencing runs such as the one shown in Figure 1c, where a single low quality cycle resulted in the presence of an unknown or low quality nucleotide in the middle of each read. After “recovery”, resulting reads can still be used by most *de-novo* assembly and SNP detection programs.

A sharp decline in the quality of the last nucleotides or the presence of undesirable prefixes may require the user to (4) trim the prefixes and/or suffixes of all the reads in the dataset. This option is available at any step of the data analysis. *Slim-Filter’s* filtration capabilities include: (5) the elimination of all reads containing odd or unknown characters; (6) the removal of low complexity reads (such as reads where a single nucleotide exhibits a proportion above a user defined threshold); (7) the exclusion of reads containing template subsequences; and (8) the interactive deletion of the most abundant reads in the dataset. Additional data manipulation features include: (9) the removal of duplicated and (10) reverse complementary reads (while maintaining their copy numbers), and (11) the sorting and storing of reads by order of ascending copy number and in the *AS* data format, which allows the user to easily view or edit the sequencing data files.

### Statistical assessment of reads

Following each step, *Slim-Filter* recalculates and visualizes the statistical characteristics of the altered dataset in a new window (Figure 2a-g). The graphical output includes: the (a) GC content and (b) entropy distributions for all reads in the dataset, as well as only the unique reads (when the copy number is not considered). It also includes (c) the average position-by-position quality and (d) proportion of each nucleotide, (e) the copy number distribution of the reads, and (f) a list of the 50 most frequent reads. These graphical outputs have been chosen to visually detect the most frequent sequencing instrument errors such as scanner calibration, quality drops due to mechanical disturbances, and sample and library preparation errors. The entropy of each individual read is calculated using the formula $E=-\sum_{i=1}^{5}p_{i}\ln(p_{i})$, where *p*_*i*_ is the proportion of each of the four considered nucleotides (A, T, C, G, and *N* for unknown characters) in each given read. A short execution trace (g) is available for each window and contains a summary of the previously applied step. The pop-up filter option (Figure 2h) opens a list of tasks that can be performed at each given step of the analysis. The “Exclude Reads Containing Subsequences” option opens a new window, allowing the user to define subsequences to be used in the filtration process. At any point during the session, the user can save a detailed step-by-step report containing all data, which can be used to re-construct the presented graphs using third-party software such as Excel, Matlab, R, etc.

## Results and Discussion

### Linux and windows versions

The core code of *Slim-Filter* is written in standard C++ and is available in both Windows and Linux versions. The *.Net* chart controls are used to display statistical properties. In case of very large input datasets and the limited amount of main memory for Windows desktop computers, performance will depend on the frequency of garbage collection performed by the Windows operating system. An estimate of the required memory for different reads’ file sizes can be found on the supplementary website.

The Linux version lacks a graphical user interface and represents a single-step execution command line application. The Linux version also requires a full set of parameters to be present in the command line.

### Future work

We see *Slim-Filter* as part of a line of Windows-based applications focused on moving basic (quality assessment, search, mapping, and *de-novo* assembly) Next Generation Sequencing data analysis tasks from specialized computational facilities to the investigator’s desktop computer. Future versions of *Slim-Filter* will support paired-end reads as well as reads of flexible sizes generated by the 454 Life Sciences GS FLX and the Ion PGM™ and Ion Proton™ (Life Technologies) Sequencing instruments.

## Conclusion

*Slim-Filter* has been developed with the goal of providing individual investigators with integrated sequencing reads analysis, curation, and manipulation capabilities. It allows the user to assess quality, collect statistics, and perform basic data manipulation tasks on a set of short reads of equal sizes. Multiple error types such as biases at the beginning of the reads, damaged sequencing cycles, sequencing of adapters, and a drop in quality scores at any position in the reads can be treated or trimmed under both Windows and Linux versions of the program. *Slim-Filter* supports the *fasta, fastq,* and *AS* file formats. Setup files for Windows 64-bit operating systems and binary files for the Linux operating system are available at http://www.bioinfo.uh.edu/Slim_Filter.

## Availability and requirements

**Project name:***Slim-Filter*

**Project home page:**http://www.bioinfo.uh.edu/Slim_Filter

**Operating system(s):** Windows 64 bit, Linux

**Programming language:** VC++/C++

**Other requirements:** Microsoft.NET Framework 3.5 or higher

**License:** GNU

## Competing interests

The authors’ declare that they have no competing interests.

## Authors’ contributions

GG has implemented the Windows and Linux versions of *Slim-Filter* and constructed the first draft of the paper. AM-A and WW analyzed various types of sequencing biases/errors and helped identify appropriate ranges for the statistical parameters to be monitored as well as the data manipulation tasks to be implemented. JJH, EB, KK, SG, and MR participated in the design, development and testing of *Slim-Filter*. YF developed the core C++ classes used in *Slim-Filter* and supervised the project. All authors have read and approved the final manuscript.
